# Clinical characteristics, diagnosis, treatment, and prognosis of Atezolizumab-induced encephalitis, aseptic meningitis or meningoencephalitis

**DOI:** 10.3389/fnhum.2025.1443463

**Published:** 2025-01-28

**Authors:** Qingzi Yan, Yixiang Hu, Xiang Liu, Hong Xia

**Affiliations:** Department of Clinical Pharmacy, Xiangtan Central Hospital, Xiangtan, China

**Keywords:** atezolizumab, PD-L1, encephalitis, immune checkpoint inhibitor, aseptic meningitis, meningoencephalitis

## Abstract

**Objective:**

Immune checkpoint inhibitors (ICIs) have revolutionized cancer treatment and expanded the range of tumor indications. However, as the usage of this medication has increased, related adverse events are increasingly being identified. Among these, Atezolizumab-induced encephalitis, aseptic meningitis, and meningoencephalitis remain poorly understood regarding clinical features. This study provides a comprehensive reference for classifying, identifying, and managing Atezolizumab-associated neurological adverse events, specifically encephalitis, aseptic meningitis, and meningoencephalitis.

**Methods:**

This study systematically collected published case reports from public databases such as PubMed, Web of Science, Wanfang Database, and CNKI Database. Two independent reviewers screened titles and abstracts and then extracted data in the included article according to the designed table and analyzed the clinical characteristics of Atezolizumab-induced encephalitis, aseptic meningitis, or meningoencephalitis.

**Results:**

A total of 17 articles were included, with 19 patients diagnosed with encephalitis, aseptic meningitis, or meningoencephalitis after Atezolizumab treatment. The most common presenting symptoms included fever, altered consciousness, fatigue, somnolence, and seizures. Diagnosis was primarily based on cerebrospinal fluid analysis, blood tests, and imaging studies, such as computed tomography (CT) scans. Treatment strategies typically involved systemic steroids, antiviral agents, antibiotics, and anti-epileptic medications, as appropriate.

**Conclusion:**

Neurological immune-related adverse events may rapidly progress and impact prognosis. Therefore, clinical practitioners should have a deep understanding of these neurological immune-related adverse events, promptly diagnose them, and provide accurate and timely treatment.

## Introduction

Atezolizumab is a humanized monoclonal antibody that targets the programmed cell death ligand (PD-L1), releasing the immune suppression mediated by PD-1/PD-L1 and thereby promoting T cells to attack tumor cells. Approved by the FDA in 2016, Atezolizumab was later authorized for the treatment of small cell lung cancer (SCLC) in China in 2020 (Johnson et al., 2022; Bagchi et al., 2021). As the use of Atezolizumab becomes more widespread, the understanding of immuno-related adverse events of Atezolizumab becomes increasingly important. However, encephalitis, aseptic meningitis, and meningoencephalitis—rare but serious immune-related adverse events—have received insufficient attention to Atezolizumab. Currently, there are no large-scale randomized controlled trials on the diagnosis and treatment of these neurological adverse events. The understanding of Atezolizumab-induced encephalitis, aseptic meningitis, or meningoencephalitis is largely based on case reports, and the clinical features remain unclear. Therefore, we conducted a summary analysis of cases of encephalitis, aseptic meningitis, or meningoencephalitis induced by Atezolizumab, aiming to provide reference experience for the diagnosis and treatment of encephalitis, aseptic meningitis, or meningoencephalitis induced by Atezolizumab.

## Methods

### Retrieval strategy

We conducted an independent search for full-text articles in both Chinese and English databases including Pubmed, Web of Science, Wanfang Database, and CNKI database to collect case reports, case series, and clinical studies related to Atezolizumab-induced encephalitis, aseptic meningitis or meningoencephalitis. The search was performed by two authors, covering publications from the launch of Atezolizumab up until January 30, 2024. The search strategy (as shown in S1) included the following key terms: ‘Atezolizumab’, ‘anti-PDL1’, ‘PD-L1 Inhibitor’, ‘PD-L1 Blockade’, ‘Programmed Death-Ligand 1 Inhibitors’, ‘immune Checkpoint Inhibitor’, ‘Immune Checkpoint Inhibition’, ‘Immune Checkpoint Blockers’, ‘Immune Checkpoint Blockade’, ‘MPDL3280A’, ‘Tecentriq’, ‘RG7446’, ‘Brain Inflammations’, ‘inflammation, Brain’, ‘Brain Inflammation’, ‘Rasmussen Syndrome’, ‘Rasmussen’s Syndrome’, ‘Encephalitis**’**, **‘**Meningitides’, ‘Pachymeningitis’, ‘Pachymeningitides.’

### Inclusion and exclusion criteria

Inclusion criteria for this study were as follows: (1) case reports documenting Atezolizumab-induced encephalitis, aseptic meningitis, or meningoencephalitis; (2) full articles written in Chinese or English.

Exclusion criteria for this study were as follows: (1) studies published by review; (2) mechanistic studies; (3) only abstract, no full-text research; (4) animal studies; (5) duplicate cases.

### Data extraction

Based on the designed table, two independent reviewers screened titles and abstracts and then extracted data from the included article. Patients’ data including gender, age, country, primary disease, medication history and concurrent medications, secondary disease diagnosis, onset time, clinical manifestations, head CT or MRI findings, Cerebral spinal fluid (CSF) analysis, treatment, and outcomes.

### Statistical analysis

Data are reported as number (percentage) or median (range). Comparisons of demographic and other features between patients presenting with encephalitis, aseptic meningitis, or meningoencephalitis were performed with Fisher’s test, the chi-squared test, and the Mann–Whitney *U* test, as appropriate.

## Results

### Basic information

**Figure 1:**
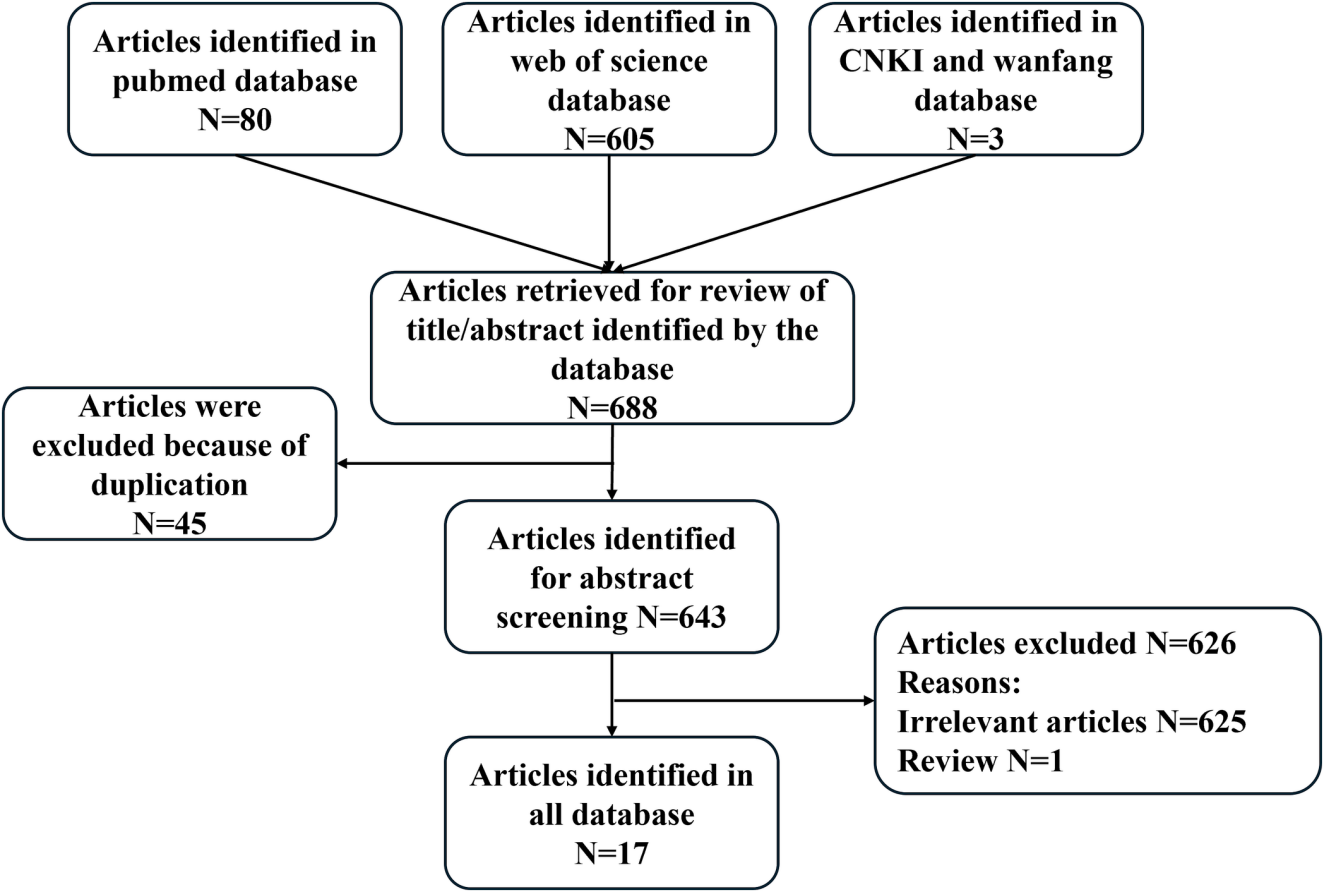
Article selection methodology.

A total of 19 patients with encephalitis, aseptic meningitis, or meningoencephalitis were selected, including 9 (47.4%) males and 10 (52.6%) females and the article selection methodology as shown in Figure 1. The basic characteristics of 19 patients are summarized in Table 1. The median age of the patients was 59 years (range 38, 78), with the majority from Japan (8, 42.1%), followed by the United States (4, 21.1%), China (3, 15.8%), Germany (1, 5.3%), Lebanon (1, 5.3%), Korea (1, 5.3%), France (1, 5.3%). Six patients received Atezolizumab treatment for non-small cell lung cancer (NSCLC), accounting for 31.6% of the cases. Five patients were treated for small cell lung cancer (SCLC), representing 26.3%. Additionally, three patients were treated for liver cancer, which constituted 15.8% of the cases. Two patients with bladder cancer were also treated with Atezolizumab, making up 10.5% of the total. Lastly, three cases received Atezolizumab treatment for breast cancer (10.5%) and one for cervical cancer (5.3%). More than 80% of cancer patients developed drug-related encephalitis, aseptic meningitis, or meningoencephalitis after 2 weeks of being treated with Atezolizumab, and a small number of patients developed drug-related encephalitis, aseptic meningitis, or meningoencephalitis after a prolonged period of treatment.

**Table 1:** Basic characteristics of the 19 patients included.

ID	Country	Age (years)	Gender	Primary disease	History of drug use or combination therapy	The first onset time after initiation of treatment	Reference
1	Germany	70	Female	Multifocal hepatocellular carcinoma with macrovascular invasion	Bevacizumab	10 days	Özdirik et al. (2021)
2	Japan	56	Male	Lung adenocarcinoma with lymph node metastasis	Right lobectomy, cisplatin plus vinorelbine, carboplatin plus nab-paclitaxel	10 days	Yamaguchi et al. (2020)
3	Japan	42	Female	HCC	Bevacizumab, lenvatinib, cisplatin	12 days	Satake et al. (2022)
4	USA	59	Female	Metastatic bladder cancer	Gemcitabine and cisplatin	12 days	Levine et al. (2017)
5	Japan	78	Male	Metastatic lung tumor of the left temporal lobe	Chemotherapy and radiation therapy	13 days	Arakawa et al. (2019)
6	Taiwan China	76	Male	HCC	Bevacizumab	14 days	Chao and Tseng (2023)
7	China	65	Female	Infiltrating ductal carcinoma	Epirubicin, cyclophosphamide, and docetaxel	10 days	Chen et al. (2022)
8	Japan	71	Female	NSCLC		14 days	Toyozawa et al. (2020)
9	Japan	55	Male	Lung adenocarcinoma		11 days	Toyozawa et al. (2020)
10	Japan	50	Male	Lung adenocarcinoma		11 days	Toyozawa et al. (2020)
11	Lebanon	38	Female	Triple-negative breast cancer	Adriamycin and Cyclophosphamide followed by Docetaxel, Carboplatin and Paclitaxel, Bevacizumab, and Capecitabine, then Eribulin.	10 days	Nader et al. (2021)
12	USA	53	Female	Cervical squamous cell carcinoma	Bevacizumab	13 days	Laserna et al. (2018)
13	USA	71	Female	SCLC with brain metastasis	Carboplatin and etoposide	4 months	Ibrahim et al. (2022)
14	Korea	49	Male	Urothelial carcinoma of the bladder		14 days	Kim et al. (2019)
15	France	48	Female	Metastatic lung adenocarcinoma		13 days	Robert et al. (2020)
16	Japan	76	Male	SCLC	Carboplatin, etoposide	5 months	Tatsumi et al. (2021)
17	Japan	66	Male	SCLC	Carboplatin and etoposide	2 months	Nakashima et al. (2022)
18	China	66	Female	SCLC	Paclitaxel carboplatin	4 days	Xiao and Yanhua (2023)
19	USA	47	Male	SCLC	Carboplatin, etoposide, denosumab		Mandal and Gosain (2021)

### Clinical symptoms

The clinical symptoms of 19 patients are summarized in Table 2. Fever (13, 68.4%), seizure (7, 36.8%), somnolence (6, 31.6%) and consciousness disorder (11, 57.9%) are the most common symptoms of encephalitis, aseptic meningitis or meningoencephalitis, and other accompanying symptoms included fatigue (4, 21.1%), mental altered (3, 15.8%), dyspnea (2, 10.5%), articular pain and sore (2, 10.5%), as well as cognition impaired specific neurological symptoms such as cognition impaired temporospatial disorientation, memory impairment and aphasia (4, 21.1%). Some patients also experienced unique symptoms including vomiting, hypothermia, headache, tachycardia, forgetfulness and irritability, dysphagia, gait disturbance, dizziness, Emotional instability, and speech confusion.

**Table 2:** Clinical symptoms of the 17 included patients.

ID	Diagnosis of secondary disease	Fever	Consciousness disorder	Fatigue	Somnolence	Seizures	Mental altered	Dyspnea	Nonspecific neurological symptoms	Articular pain and sore	Others
1	Encephalitis	√		√	√	√		√	√		
2	Encephalitis	√	√		√						
3	Encephalitis	√	√			√					
4	Encephalitis	√	√	√		√					Vomiting
5	Encephalitis	√	√		√						
6	Encephalitis		√						√		hypothermia
7	Encephalitis		√		√						
8	Aseptic Meningitis	√	√								
9	Aseptic Meningitis	√	√								
10	Aseptic Meningitis	√	√							√	
11	meningoencephalitis	√	√			√					
12	Encephalitis				√	√	√				Headache, tachycardia
13	Encephalitis		√								
14	Encephalitis	√		√	√	√					
15	Meningitis	√					√	√	√	√	
16	Encephalitis										Forgetfulness and irritability
17	Encephalitis								√		dysphagia, gait disturbance
18	Encephalitis	√				√	√				Dizziness, Emotional instability, speech confusion
19	Encephalitis	√		√							

### Laboratory testing

In routine laboratory tests, the routine laboratory tests of some patients have not been reported, the patient’s main manifestations were elevated CRP, lymphocytes, and leukopenia. Most patients (17, 89.5%) underwent laboratory tests including cerebrospinal fluid analysis (CFS) was reported in 17 of 19 patients, mainly manifested as increased cell count (11, 57.9%), protein level (15, 78.9%) and glucose level (9, 47.4%). Moreover, CFS analysis showed no malignant tumor cells, no central nervous system infection, and no paraneoplastic inflammation, and CSF culture for negative for bacterial, fungal, viral, and parasite (12, 63.1%). Paraneoplastic antibodies were detected in 10 patients, with 5 patients testing negative and the paraneoplastic antibodies of the other 5 patients’ antibody profiles included anti-CRMP5, Anti-SOX1, Anti-Hu, and Anti-Zic4 positivity.

### Imaging examination

Seventeen patients underwent head CT or MRI, with eleven cases (11, 57.9%) showing no significant abnormalities on the imaging studies, while only 6 patients exhibited abnormal brain imaging. Four of the six patients (4, 21.1%) exhibited diffuse leptomeningeal hyperintensities enhancement. One patient (1, 5.3%) showed a small subdural hematoma and subarachnoid hemorrhage at the right parietal region. Another patient (1, 5.3%) showed multiple patchy T2 hyperintensities in the bilateral cerebellar hemisphere, vermis of the cerebellum, bilateral frontal lobe, temporal lobe, parietal lobe, and occipital cortex.

### Treatment and prognosis

The treatment and prognosis of 19 patients with encephalitis, aseptic meningitis, or meningoencephalitis are summarized in Table 3. All patients (19, 100%) received systemic Steroids after being diagnosed with encephalitis, aseptic meningitis, or meningoencephalitis. Among these, eight patients (8, 42.1%) were also treated with antiviral, antibacterial, antifungal, and other antimicrobial agents. Two patients (2, 10.5%) administered intravenous immunoglobulins (IVIG). Additionally, five patients (5, 26.3%) were treated with levetiracetam, valproic acid, and other antiepileptic drugs. The symptoms of encephalitis, aseptic meningitis, or meningoencephalitis in 17 patients were improved after treatment. Two patients (2, 10.5%) did not show improvement after treatment; one of the two patients continued to experience fever and elevated C-reactive protein (CRP) levels despite antibiotic administration, while another’s condition deteriorated, resulting in cardiac and respiratory arrest.

**Table 3:** Laboratory examination, treatment, and prognosis of 19 patients included.

ID	Laboratory testing	CT and MRI	Cerebral spinal fluid (CSF) analysis	Treatment	Outcome
1	Elevated CRP, and decreased leucocytes and lymphocytes	No abnormalities	Elevated leucocyte count; increased protein level, no tumor cells, no infectious, autoimmune, or paraneoplastic inflammation	Methylprednisolone, anti-infective therapy, dilatative tracheostomy	Improved
2	NA	No abnormalities	Elevated cell count, protein, glucose, IL-6, negative for bacterial and viruses cultures	Steroid	Improved
3	NA	No abnormalities	Elevated WBC, protein, and glucose; negative for bacterial and fungal and viruses cultures	Methylprednisolone, Levetiracetam	improved
4	Electrolyte, glucose, creatinine, and liver function tests were unremarkable	No abnormalities	Elevated glucose and protein, negative for bacterial and fungal CSF cultures	Dexamethasone, levetiracetam, anti-infective therapy	Improved
5	NA	No abnormalities	Elevated cell count and protein, negative for bacterial and virus cultures	Methylprednisolone, ceftriaxone, acyclovir	Improved
6	Elevated CRP, and lactate acid	A small subdural hematoma and subarachnoid hemorrhage at the right parietal region	Elevated RBC and WBC, protein and albumin levels; negative for bacterial, fungal, viral, and tuberculous cultures	Methylprednisolone	Improved
7	Decreased lymphocyte, haemoglobin level, and blood oxygen saturation; elevated D-dimer	Multiple patchy T2 hyperintensities in the bilateral cerebellar hemisphere, vermis of the cerebellum, bilateral frontal lobe, temporal lobe, parietal lobe, and occipital cortex.	NA	Dexamethasone	Aggravation
8	NA	No abnormalities	Elevated protein	Methylprednisolone	Improved
9	NA	No abnormalities	Elevated protein	Methylprednisolone	Improved
10	NA	Multiple abnormal enhancements	Increased monocytes cells, protein; negative for bacteria cultures	methylprednisolone, levetiracetam	Improved
11	Leukopenia	Moderate diffuse leptomeningeal enhancement	Elevated WBC count, protein, lymphocytosis, glucose; negative for microbe culture	Broad-spectrum antibiotics and antivirals, dexamethasone	Improved
12	NA	No abnormalities	Elevated leukocytes, neutrophils, RBC, proteins, glucose; no malignant cell, negative for microbe culture	Antibiotics, corticosteroids, lorazepam levetiracetam	Improved
13	NA	No abnormalities	Elevated lymphocytic pleocytosis, WBC, lymphocytes, protein, glucose; no malignant cells, negative for microbe culture	Steroids	Improved
14	Elevated CRP	Diffuse leptomeningeal enhancement	Elevated WBC, no malignant cells, CSF culture negative for bacterial, fungus, tuberculous, and viral culture	Antibiotics, methylprednisolone, IVIG	No improvement
15	Elevation of CRP, anemia, lymphopenia, hyponatremia	No abnormalities	Elevated protein and glucose, no malignant cells, negative for bacterial culture	Antibiotics, dexamethasone	Improved
16	Anti-CRMP5 antibody positive	T2hyperintensity in the bilateral striatum	Normal glucose level, elevated protein, and cell count	steroid	Improved
17	Anti Hu and anti-Zic4 antibodies positive	A high-intensity area in the bilateral temporal lobes	NA	Steroid, IVIG	Improved
18	Granulocytopenia, anti-SOX1 antibodies positive	No abnormalities	Elevated protein and glucose	Antibiotics, valproic acid, Methylprednisolone	Improved
19	Anti-Hu antibodies positive	NA	NA	Prednisone	Improved

NA, not available. IVIG, Intravenous Immunoglobulin.

## Discussion

Most patients experiencing Atezolizumab-induced encephalitis, aseptic meningitis, or meningoencephalitis showed improvement in symptoms after discontinuation of Atezolizumab and appropriate treatment. Therefore, a graded assessment of adverse reactions should be conducted to determine whether discontinuation of Atezolizumab is necessary. Patients with lower-grade adverse reactions (G1, G2) typically do not require discontinuation of treatment (Schneider et al., 2021). This study suggests that Atezolizumab-induced encephalitis, aseptic meningitis, or meningoencephalitis typically manifests in most patients within approximately 2 weeks of initiating Atezolizumab therapy. However, a minority of patients may develop central nervous system adverse reactions after prolonged use of Atezolizumab. The onset time of Atezolizumab-induced encephalitis, aseptic meningitis, or meningoencephalitis varied significantly, ranging from 1 day to 5 months after treatment initiation, highlighting the unpredictability of immune-related adverse events. Reports indicate that the median latency period from the initiation of immune checkpoint inhibitor (ICI) therapy to the occurrence of these events was 8 weeks (Fan et al., 2020).

Drug-induced immune-related encephalitis, aseptic meningitis, or meningoencephalitis should be suspected when patients experiencing epileptic seizures with abnormal mental state, speech disorder, dystonia, or various forms of tonic–clonic seizures, fever, headache, joint pain or neurological abnormalities during the use of Atezolizumab (Watanabe et al., 2019). However, it is important to distinguish Atezolizumab-induced encephalitis, aseptic meningitis, or meningoencephalitis from other types of encephalitis, as the clinical features of infectious encephalitis closely resemble those of drug-induced encephalitis, aseptic meningitis, and meningoencephalitis (Seki et al., 2022). Common symptoms of infectious and immune encephalitis, as well as aseptic meningitis or meningoencephalitis encompass fever and altered consciousness. In addition, patients with immune-related encephalitis, aseptic meningitis, or meningoencephalitis, as well as those with infectious encephalitis, may present with seizures, drowsiness, coma, and changes in mental state (Meyfroidt et al., 2020). Therefore, CSF analysis is essential for ruling out other etiologies. In cases of immune encephalitis, CSF analysis typically shows an elevated lymphocyte count, increased protein levels, and decreased glucose levels. Furthermore, CSF cultures from patients with immune encephalitis, aseptic meningitis, or meningoencephalitis generally do not detect any microorganisms.

In addition to CFS and culture, imaging examinations may help to distinguish between Atezolizumab-induced encephalitis, aseptic meningitis, or meningoencephalitis and other forms of encephalitis. For more than half of patients with Atezolizumab-induced encephalitis, aseptic meningitis, or meningoencephalitis, imaging findings are normal. However, a small proportion of patients showed brain imaging abnormalities, including diffuse leptomeningeal hyperintensities enhancement, subdural hematoma, subarachnoid hemorrhage, and multiple patchy T2 hyperintensities in the bilateral cerebellar hemisphere, vermis of the cerebellum, bilateral frontal lobe, temporal lobe, parietal lobe, and occipital cortex.

Regarding the treatment of drug-induced immune encephalitis, aseptic meningitis, or meningoencephalitis, all patients receive high-dose steroid therapy, two patients are also treated with IVIG, and partial of patients also receive empirical antibiotics and antiviral therapy, as well as supportive treatments such as fluid supplementation, plasma exchange, and tracheostomy. The majority of patients presenting with Atezolizumab-induced encephalitis, aseptic meningitis, or meningoencephalitis exhibit significant symptomatic improvement following treatment. This includes the restoration of language faculties, a reduction in fever, and the mitigation of other symptoms such as seizures and fatigue.

The exact pathophysiology of drug-induced autoimmune encephalitis, aseptic meningitis, or meningoencephalitis is not yet fully clear. ICIs may induce encephalitis, aseptic meningitis, or meningoencephalitis through multiple pathways (Wu et al., 2019). Combining PD-L1 with PD-1 can induce T cell apoptosis, dysfunction, and exhaustion, inhibiting the activation, proliferation, and anti-tumor function of tumor antigen-specific CD8 + T cells, inhibition of PD1 and CTLA4 can stimulate antibody production, thereby leading to antibody-mediated autoimmune diseases (Llovet et al., 2022). Atezolizumab selectively binds to PD-L1, blocks the PD-1/PD-L1 signaling pathway, enhances the recognition of tumor cells by immune cells T cells in the body, and kills tumor cells after T cell activation (Huppert et al., 2020). However, after tumor cell destruction, intracellular antigens are released into the systemic environment, which may trigger the immune system to produce aberrant antibodies, potentially precipitating autoimmune conditions such as autoimmune encephalitis, aseptic meningitis, or meningoencephalitis (Fan et al., 2020). Furthermore, immune checkpoint inhibitors (ICIs) can elicit immune responses against shared self-antigens found in both tumor and neural tissues. Patients experiencing ICI-related toxicities may exhibit a pre-existing susceptibility before the initiation of immunotherapy, potentially due to the presence of anti-neuronal antibodies. The target antigens of immune checkpoint inhibitors (ICIs), namely PD-1, PD-L1, and CTLA-4, are also expressed on cells within the nervous system. This expression can lead to complement-dependent or cell-mediated cytotoxic reactions following ICI engagement, thereby potentially inducing adverse neuronal reactions (Farina et al., 2024; Velasco et al., 2021) (Figure 1).

## Conclusion

**Figure 2:**
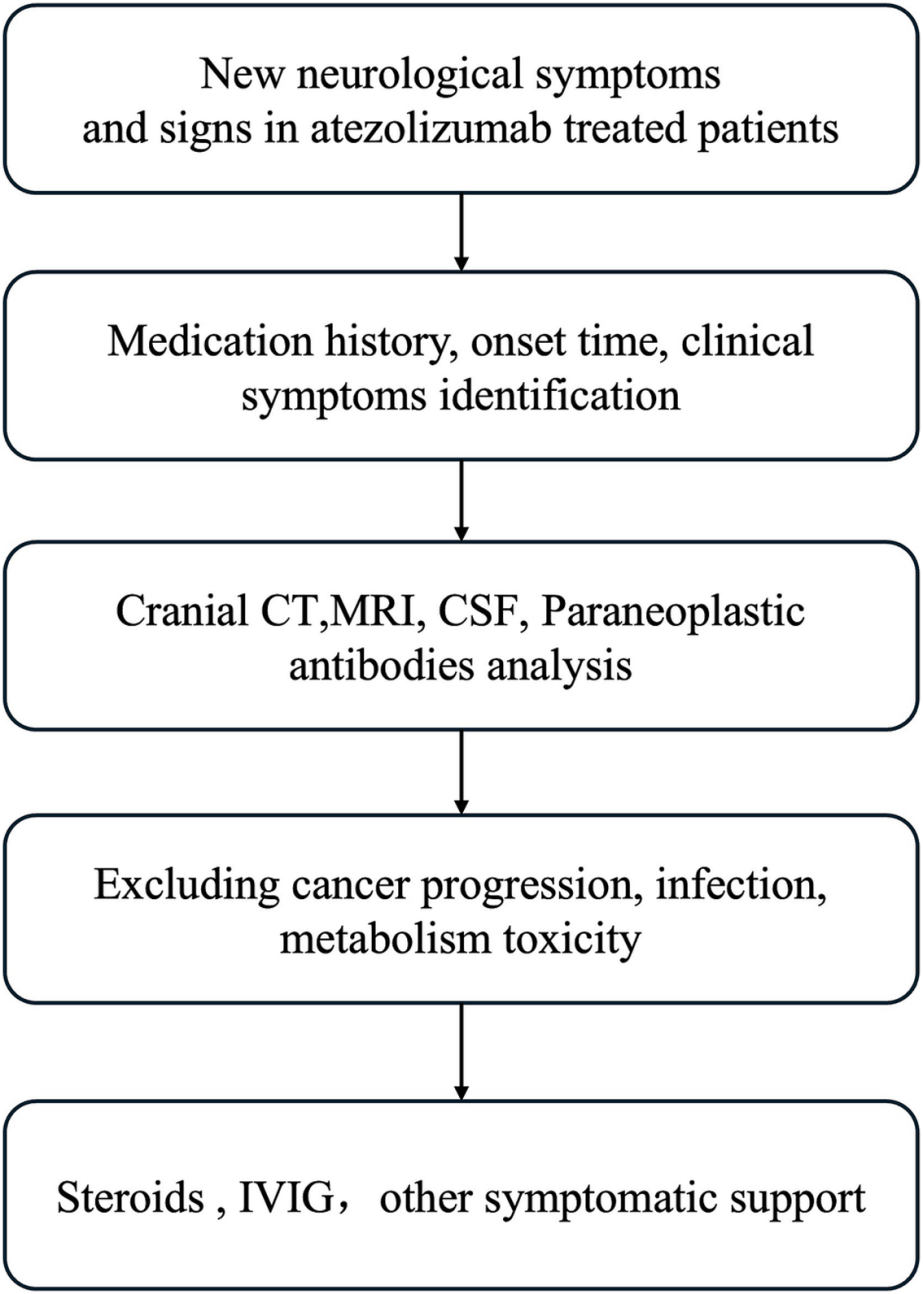
The diagnostic process of immune-related adverse effects.

Neurological immune-related adverse events, especially autoimmune encephalitis, aseptic meningitis, or meningoencephalitis, are rare but serious complications. The initial nonspecific symptoms of neurological immune-related adverse events (irAEs) present a significant challenge for diagnosis. Moreover, these irAEs can progress rapidly, significantly impacting patient prognosis. Therefore, healthcare providers must have a thorough understanding of these conditions, enabling them to make timely and accurate diagnoses and administer appropriate interventions. This study presents a summary of a recommended diagnostic workflow for reference purposes as shown in Figure 2.
